# Investigation of pharmacological mechanism of natural product using pathway fingerprints similarity based on “drug-target-pathway” heterogenous network

**DOI:** 10.1186/s13321-021-00549-5

**Published:** 2021-09-20

**Authors:** Feifei Guo, Chunhong Jiang, Yujie Xi, Dan Wang, Yi Zhang, Ning Xie, Yi Guan, Fangbo Zhang, Hongjun Yang

**Affiliations:** 1 Institute of Chinese Materia Medica, China Academy of Chinese Medical Sciences, Beijing, China; 2 Joint Institute of Virology (Shantou University and The University of Hong Kong), Shantou University Medical College, Shantou, China; 3 Tianjin University of Traditional Chinese Medicine, Tianjin, China; 4 State Key Laboratory of Proteomics, Beijing Proteome Research Center, National Center for Protein Sciences-Beijing (PHOENIX Center), Beijing Institute of Lifeomics, Beijing, China; 5 State Key Laboratory of Innovative Natural Medicine and TCM Injections, Ganzhou, China; 6 Beijing Key Laboratory of Traditional Chinese Medicine Basic Research on Prevention and Treatment for Major Diseases, Experimental Research Center, China Academy of Chinese Medical Sciences, Beijing, China

**Keywords:** Natural products, Andrographolide derivative, Pathway fingerprints, “Drug-target-pathway” network, Anti-inflammatory

## Abstract

Natural products from traditional medicine inherit bioactivity from their source herbs. However, the pharmacological mechanism of natural products is often unclear and studied insufficiently. Pathway fingerprint similarity based on “drug-target-pathway” heterogeneous network provides new insight into Mechanism of Action (MoA) for natural products compared with reference drugs, which are selected approved drugs with similar bioactivity. Natural products with similar pathway fingerprints may have similar MoA to approved drugs. In our study, XYPI, an andrographolide derivative, had similar anti-inflammatory activity to Glucocorticoids (GCs) and non-steroidal anti-inflammatory drugs (NSAIDs), and GCs and NSAIDs have completely different MoA. Based on similarity evaluation, XYPI has similar pathway fingerprints as NSAIDs, but has similar target profile with GCs. The expression pattern of genes in LPS-activated macrophages after XYPI treatment is similar to that after NSAID but not GC treatment, and this experimental result is consistent with the computational prediction based on pathway fingerprints. These results imply that the pathway fingerprints of drugs have potential for drug similarity evaluation. This study used XYPI as an example to propose a new approach for investigating the pharmacological mechanism of natural products using pathway fingerprint similarity based on a “drug-target-pathway” heterogeneous network.

**Supplementary Information:**

The online version contains supplementary material available at 10.1186/s13321-021-00549-5.

## Introduction

More than half of new chemical entities from 1981 to 2010 were derived from natural products directly or indirectly [1]. Natural product-based drugs are often identified by phenotypic assays, and deconvolution of their MOA can be challenging and time-consuming [2, 3]. The approved drugs with clear MoA provide a benchmark for natural product to follow. Comparison with approved drugs with clear mechanisms can help to explore the exact MoA of a natural product. As a natural product, andrographolide is the main active ingredient of *Andrographis paniculate*, which makes up about 4%, 0.8 ~ 1.2% and 0.5 ~ 6% in dried whole plant, stem and leaf extracts respectively [4–7]. And *Andrographis paniculate* was used as an herbal medicine in both traditional Indian and Chinese medicine (where it is known as kalmegh and chuanxinlian, respectively) [8], which also exhibit anti-inflammatory activity that is commonly attributed to andrographolide [9]. Xiyanping injection (XYPI) is a CFDA-approved drug that consists of andrographolide derivatives, including water-soluble sulfonated andrographolide [10]. In the clinic, Glucocorticoids (GCs) and non-steroidal anti-inflammatory drugs (NSAIDs) both have anti-inflammatory effects with distinct MoA [11, 12]. Our study attempts to confirm the mechanism of XYPI (andrographolide derivatives) using both NSAIDs and GCs as references. Commonly used GCs and NSAIDs were chosen as positive controls to analyze the similarities and differences with XYPI [12].

Drugs without common targets can also exert similar therapeutic effects on the same disease because different targets participate in the same pathway or interact with each other, which is closely associated with the pathological process [13]. Therefore, in addition to structure-based and target-based viral screening methods, a pathway-based approach was been applied for nature product-based drug discovery [13, 14] and repositioning [15]. Bayesian sparse factor analysis model was used to identify the target biological pathways for drugs with unclear mechanism of action based on the joint analysis of gene expression and drug sensitivity profiles measured on the same set of cell lines [16]. Besides that, matrix decomposition-based machine learning methods have been used for identification of drug-pathway associations [17–19]. Gene2Drug, a computational method for drug repositioning, was developed to assesses the drug impact of transcription of pathway [20]. Fukuoka et al. proposed a drug repositioning method based on a protein–protein interaction (PPI) network of two diseases and the similarity of the drugs [21].

However, known “natural product-pathway” associations discovered by biological experiments are too less. Only Connectivity Map (cMap) has been employed to predict potential drug-pathway association which collect genomewide transcriptional expression data form cell lines after small molecules treatment [22, 23]. But few natural products were included in cMap, and discovery of “natural product-pathway” associations by experimental methods is time consuming and laborious. Fortunately, “drug-target-pathway” heterogenous network is easily constructed based on prediction of drug-target interactions and gene-pathway associations from pathway database (including KEGG Pathway, Reactome, WikiPathways, MSigDB) [24–27]. As a semi-structured representation method, “drug-target-pathway” heterogeneous information network (HIN) is an effective tool for integrating information, which can fuse three types of objects (including drug, target, pathway) and two semantics relationships between them (drug-target interactions and target-pathway association) via multiple social network platforms. In HIN, two drug objects can be connected via different semantic path, which are defined as meta-paths. The metapath not only characterizes the semantic relationship between objects but also extracts feature information between objects. The metapath “drug-target-pathway-target-drug” of two drugs was considered to describe the linkage between two drugs. The pathway fingerprint similarity of two drugs was measured based on a “drug-target-pathway” heterogeneous network using the Meta Path-based similarity search method-PathSim, which has been applied to recommendation systems in social networks [28]. And a casual heterogenous network (drug-target-pathway-gene-disease) was applied to discover new positioning of existing drugs [29]. And In our previous study, active compounds from TCM were predicted and validated by pathway-based similarity search method (PathSim) based on “drug-target-pathway” HIN [14]. In the future, heterogeneous network would be widely used in exploration of the exact MoA of natural product as a tool.

In this study, we proposed an approach using pathway fingerprint similarity based on a “drug-target-pathway” heterogeneous network to explore the mechanisms of natural drugs XYPI using NSAIDs and GCs as reference agents. A heterogeneous network similarity algorithm used for social networks was applied to investigate the pathway fingerprint similarity between drugs in the heterogeneous "drug-target-pathway" network and to predict the drug-pathway associations of XYPI. The results indicate that XYPI may have similar MoA with NSAIDs, neither than GCs. To validate the prediction result, LPS-induced macrophage activation model was applied and transcriptome of drug treatment to model were launched to investigate the transcriptome expression pattern after drug treatment, which can validate potential drug-pathway association of XYPI based on experiment data. The drug-pathway association predicted based on HIN is consistent with association from experiment data which indicate that XYPI has similar MoA with NSAIDs, and pathway-based approach using “drug-target-pathway” heterogeneous network is promising for investigating the pharmacological mechanism of natural products.

## Methods

### Construction of “drug-target-pathway” heterogeneous information network

#### Prediction of drug-target interactions of drugs

Three types of drug-target interactions of drugs (XYPI and positive/negative controls) were used to construct the target profile for drug. STITCH provides the drug-target interactions based on literature mining [30], PubChem provides the drug-target interactions based on bioactivity tests in bioassays [31, 32] and BATMAN-TCM predicts drug-target interactions based on structure and function annotation (all downloaded in Jan 2018) [33]. For a query drug-target interaction, a combined confidence score (*CS*) was applied to comprehensively evaluate the probability of a drug-target interaction from the three types of resources same like the combined score of protein–protein interactions in STRING [34], which was calculated as follows:

1 $$CS = { 1 } - \, \left( {{1 } - P_{stitch} } \right) \, \times \, \left( {{1 }{-}P_{pubchem} } \right) \, \times \, \left( {{1 } - P_{batman} } \right)$$

*P*_*stitch*_ represents the probability of drug-target interaction from STITCH, *P*_*pubchem*_ represents the probability of drug-target interaction from PubChem Bioassay (1 = active, 0 = inactive, 0.5 = inconclusive or unspecific), and *P*_*batman*_ represents the probability from BATMAN-TCM. In subsequent analysis, the drug-targets interactions with *CS* > 0.4 were selected as interactions with higher reliability for analysis.

Based on the similar structure and activities, drug-target interactions of four compositions of XYPI were combined in to one dataset to present XYPI. If there are common targets between four compositions, largest confidence score of same target was used as the final confidence score of drug-target interactions. For drug-target interactions of XYPI, using "Xi yan ping Injection”, "Andrographolide sulfonates" and "Andrographolide" as keywords to mine the medical literature abstracts from PubMed from 1950 to 2015, related genes mentioned in the literature were excavated based on sentence co-occurrence. Combining targets based on literature mining, targets predicted by BATMAN-TCM, and targets extracted from PubChem Bioassay, potential targets of XYPI were kept with combined score > 0.4.

#### Associations of target-pathway from pathway database

Associations of target-pathway were obtained from three pathway databases, including Gene Ontology (GO) [35], Reactome [25], and WikiPathways [27]. Target-pathway associations from GO were annotated by biological processes term in Gene ontology annotation (GOA, downloaded in Aug, 2021). Target-pathway associations from Reactome and WikiPathways were downloaded in Jun, 2021.

#### Construction of “drug-target-pathway” heterogeneous information network

“Drug-target-pathway” heterogeneous information network consists of three type of objects (including drug, target, pathway) and two semantics relationships between them (drug-target interactions and target-pathway associations) mentioned above.

### Hierarchical clustering of drugs based on target similarity

The target similarity between two drugs was measured based on comprehensive targets of the compounds. For example, $T_{a}$ represents target space of drug $a$, and $T_{b}$ represents target space of drug $b$. The similarity score $S_{a,b}$ for the similarity of target space of drug $a$ and $b$ was calculated as follows:

2 $$S_{a,b} = \frac{{T_{a} \cap T_{b} }}{{T_{a} \cap T_{b} }}$$

The hierarchical clustering of N compounds was executed by the R package hClust [36] based on the target similarity matrix of compounds.

GCs and NSAIDs were selected as positive controls which are representative anti-inflammatory drugs and three types of negative control without anti-inflammatory activities include H2 receptor antagonists, antidepressants, and 5-HT_3_ receptor antagonists. Only GCs with similar targets profile were included in further analysis which was cluster together into one cluster based on the hierarchical clustering of target similarity, same as the NSAIDs and three types of negative control to make these drugs representative for their MoA respectively.

### Hierarchical clustering of drugs based on similarity of pathway fingerprints from a “drug-target-pathway” heterogeneous network

The pathway fingerprint similarity of two drugs was measured based on a “drug-target-pathway” heterogeneous network using the PathSim method, which has been applied to recommendation systems in social networks [28]. Under the metapath framework, PathSim was developed to find similar objects sharing metapath in the network (e.g., find drugs with similar pathway descriptions) and to measure the similarity of objects based on metapath. Using “drug-target-pathway” network as an example, the first definition is metapath which is a path between two drugs in the form of ‘drug $x$-> target $x$-> pathway $x$ < -target $y$ < -drug $y$’. Here we use $P$ to represent all the metapath between all drugs in the “drug-target-pathway” heterogeneous network.

The second definition is the similarity of pathway fingerprints of two drugs (drug $x$, drug $y$), which is

3 $$s\left( {x,y} \right) = \frac{{2 \times \left\{ {p_{x\sim y} :p_{x\sim y} \in P} \right\}}}{{\left\{ {p_{x\sim x} :p_{x\sim x} \in P} \right\} + \left\{ {p_{y\sim y} :p_{y\sim y} \in P} \right\}}}$$

where $p_{x\sim y}$ is the path instance between drug $x$ and drug $y$, and $\{ p_{x\sim y} \}$ is the number of path instance between drug $x$ and drug $y$, $\{ p_{x\sim x} \}$ is the number of path instance between drug $x$ itself, $\{ p_{y\sim y} \}$ is the number of path instance between drug $y$ itself. The hierarchical clustering of N compounds was executed by the R package hClust [36] based on the pathway fingerprints similarity matrix of compounds.

### Prediction of drug-pathway associations of XYPI from metapath in heterogeneous network

The drug most similar to XYPI can be selected based on the result of hierarchical clustering of drugs using the heterogeneous network, whose drug-pathway association may the possible pathways of XYPI. By extracting metapath between XYPI and the most similar drug from of "drug-target-pathway" network, according to the transcriptome data of the most similar drugs in LINCS, the regulatory direction of the most similar drugs on the pathway is calculated to predict the direction of action of XYPI on the pathway.

### Validation of dug-pathway associations retrieved from genome-wide transcriptome data on macrophage inflammation model

To validate the prediction result, LPS-induced macrophage activation model was applied and transcriptome of drug treatment to this model were launched to investigate the transcriptome expression pattern after drug treatment.

#### LPS-induced RAW264.7 mouse macrophage inflammation model

*Chemicals and reagents* Trypsin–EDTA digestion solution (Beijing Solable Technology Co., Ltd.),dimethyl sulfoxide (DMSO), LPS (CST), fetal bovine serum (Gibco, New Zealand), a penicillin–streptomycin double antibody (Beijing Solable Technology Co., Ltd.), DMEM culture medium (Gibco, New Zealand), and a mouse IL-6 ELISA kit (CUSABIO, Wuhan, China) were used. The RAW264.7 mouse macrophage cell line was purchased from the Cell Resources Center of the Institute of Basic Medical Sciences, Chinese Academy of Medical Sciences, and frozen in liquid nitrogen for use. A ZD-420 electric thermostatic water bath, a Multiskan Ascent microplate reader (Thermo Electron, United States), a BS224S electronic balance (Serdulis in Germany), a Napco5410 carbon dioxide incubator (American NAPCO), a DMIL inverted microscope (Germany LEICA), an optical microscope (Olympus), a program storage box, an ultraclean platform were also used.

*Cell culture and experimental procedure* Normal RAW246.7 cells were cultured in DMEM culture medium containing a final concentration of 10% fetal bovine serum and 100,000 U/L penicillin and placed in a cell incubator with 5% CO_2_ and a temperature of 37 °C. The medium was changed once every 48–72 h according to the cell growth. After the cells had grown to 70% ~ 80% confluence, they were digested with 0.25% trypsin–EDTA and centrifuged to separate the cells. The cells were then subcultured by passage once every 6 days and frozen in liquid nitrogen for later use. The total number of inoculated cells was 2 × 10^4^ per well, and the plates were placed in a cell incubator containing 5% CO_2_ at 37 °C. After 24 h of adherence, the cell culture solution was discarded, and 100 μl of DMEM culture medium containing a final concentration of 0.5 to 5 μg/ml LPS was added for inflammatory stimulation. After 48 h of treatment, the cell supernatant in each group of wells was collected. Interleukin (IL)-6 concentrations were determined using Duo Set ELISA Kits (CUSABIO, Wuhan, China). The experiment was conducted following the manufacturer’s instructions. And the morphological differences between cells in each group were observed with a microscope.

RAW264.7 cells (5 × 10^5^ cells/well in a 6-well plate) were pretreated with or without XYPI, ketoprofen, or prednisolone for 24 h and then incubated with LPS for 48 h. Cells lysates were prepared with RIPA lysis buffer and protease inhibitors (Solarbio, Beijing, China), and centrifuged at 14,000*g* for 15 min at 4 °C. Then protein samples were completed for western assay.

The statistical significance of differences between two groups was determined by an unpaired Student's t-test. The results were considered statistically significant when the p value was less than 5%. ELISA kits were used to detect the levels of the inflammatory factor IL-6 in the cell supernatant. Data are expressed as the mean using a t-test, with P < 0.05 indicating statistical significance.

#### RNA extraction, library construction and sequencing for genome-wide transcriptome data

Total RNA was extracted with TRIzol Reagent (Thermo Fisher, USA) according to the manufacturer’s instructions, and RNA integrity was assessed using the RNA Nano 6000 Assay Kit and the Bioanalyzer 2100 system (Agilent Technologies, CA, USA). Three biological replicates were used. A total amount of 3 μg RNA per sample was applied for library construction. Briefly, sequencing libraries were generated by using the NEBNext® Ultra™ RNA Library Prep Kit for Illumina® (NEB, USA) according to the instructions. First, poly-T oligo-bound magnetic beads were applied to purify mRNA before RNA fragmentation was carried out. Then, random hexamer primers and M-MuLV Reverse Transcriptase (RNase H-) were used to synthesize first-strand cDNA, and DNA Polymerase I and RNase H were used to synthesize second-strand cDNA. After that, PCR was performed to enrich the cDNA template following adenylation of the 3’ ends of the DNA fragments and ligation of the adapters. The PCR products were purified (AMPure XP system), and the Agilent Bioanalyzer 2100 system was used to evaluate library quality. The AcBot Cluster Generation System was applied for clustering of the index-coded samples by using TruSeq PE150 Cluster Kit v3-cBot-HS (Illumina). Then, the sequencing library was sequenced on an Illumina HiSeq 4000 platform, and 150 bp paired-end reads were generated. This whole experiment was conducted at Novogene Bioinformatics Technology Co., Ltd. (Beijing, China).

The EdgeR package was used for RNA-SEQ differential gene expression analysis [37], and the resulting P-values were adjusted using the Benjamini and Hochberg’s approach for controlling the false discovery rate (FDR). Gene with a FDR <  = 0.1 and fold change >  = 1.5 were assigned as differentially expressed. The principal component analysis (PCA) method was used to visualize the clustering of samples, as it can intuitively observe the clustering of samples in the experimental group and the control group. PCA analysis and visualization was executed by the R package FactoMineR [38]. Hierarchical clustering of differentially expressed genes was generated by hClust package [36].

#### Transcriptome data analysis to build drug-pathway associations

The differentially expressed genes can be found via transcriptome data after drug treatment. According to the predicted pathways regulated by XYPI based on HIN, the transcriptome data is used to verify the regulatory direction of XYPI on pathways. The ratio of up-regulated genes in pathway after drug treatment was used to evaluate the direction of drug regulation on pathway. Hierarchical clustering of differentially expressed genes in pathway was generated by hClust package [36].

## Results

### Potential Target prediction for XYPI

**Fig. 1:**
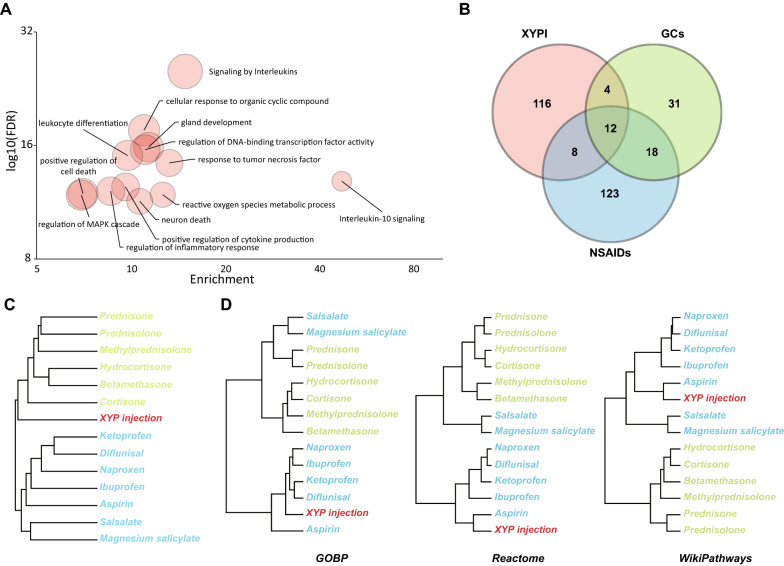
Comparison of targets of XYPI, GCs and NSAIDs. **A** Bubble chart of functional enrichment of potential XYPI targets. The X axis is the enrichment score for each term using the whole genome as background, the Y axis is the negative log10(FDR), and the node size is related to the targets matched in this term. **B** Venn diagram of the drug targets of XYPI, GCs and NSAIDs. **C**, **D** Hierarchical clustering of XYPI, GCs (green) and NSAIDs (blue) based on target similarity (**C**) and pathway fingerprint similarity based on GOBP, Reactome, WikiPathways (**D**)

By integrating the confidence scores of targets from different resources, 140 targets of XYPI with a combined confidence score greater than 0.4 were retained for subsequent analysis. Based on the functional enrichment of the integrated targets by metascape, XYPI may be involved in regulation of the inflammatory response, leukocyte differentiation, ROS metabolic process, etc. (Fig. 1A). The above results indicate that research on XYPI should focus on immune and inflammation-related pathways.

### XYPI has targets similar to those of GCs but pathway fingerprints similar to those of NSAIDs

There were 140 targets of XYPI (XYPI), 65 common targets of GCs (target for at least two GCs) and 161 common targets of NSAIDs (target for at least two NSAIDs) whose confidence scores were greater than 0.4. To investigate the similarity of XYPI with GCs and NSAIDs, Venn diagram of XYPI, GCs and NSAIDs showed that there were 12 common targets of XYPI, GC and NSAIDs, and XYPI had common targets with both GC and NSAIDs (Fig. 1B). To evaluate the target similarity between XYPI and different GCs and NSAIDs separately, unsupervised hierarchical clustering of XYPI, 6 glucocorticoids and 7 NSAIDs based on target similarity was applied. The clustering result showed that 6 glucocorticoids clustered with each other, as well as NSAIDs (Fig. 1C), which indicate that GCs have a high similarity with each other at the target level, as NSAIDs do. However, GCs and NSAIDs have relatively different target spaces, which may be due to the different MoA by which they exert anti-inflammatory effects. XYPI clustered with glucocorticoids, which shows that the targets of XYPI are more similar to those of glucocorticoids than those of NSAIDs.

After investigating the similarity of the pathway fingerprints of XYPI, GCs and NSAIDs, the unsupervised clustering results showed that XYPI is more similar to NSAIDs at pathway level, which was contrary to the previous target-based clustering results. This result was confirmed by three different pathway datasets, including Biological Process terms in GO (GOBP), Reactome and WikiPathways (Fig. 1D). For target-pathway association from GOBP, although seven NSAIDs were separated into two clusters, XYPI was still clustered with 5 NSAIDs based on pathway fingerprint similarity. Besides that, to assesses the robustness of method, three types of drugs without anti-inflammatory activity were chosen as negative controls. XYP injection still cluster with NSAIDs after negative control added in the hierarchical clustering of drugs based on pathway fingerprints similarity which is consistent with previous result without negative control (Additional file 1: Figure S1, Additional file 2: Figure S2). The results based on different pathway datasets and negative controls were consistent, which reflect the robustness of pathway-based drug similarity search methods and reliability of the result. To demonstrate the topic simply, we choose GOBP as pathway annotation database for following analysis which is very commonly used for Gene functional annotation.

### XYPI has pathway fingerprints similar to those of NSAIDs in terms of immune and inflammatory pathways

**Fig. 2:**
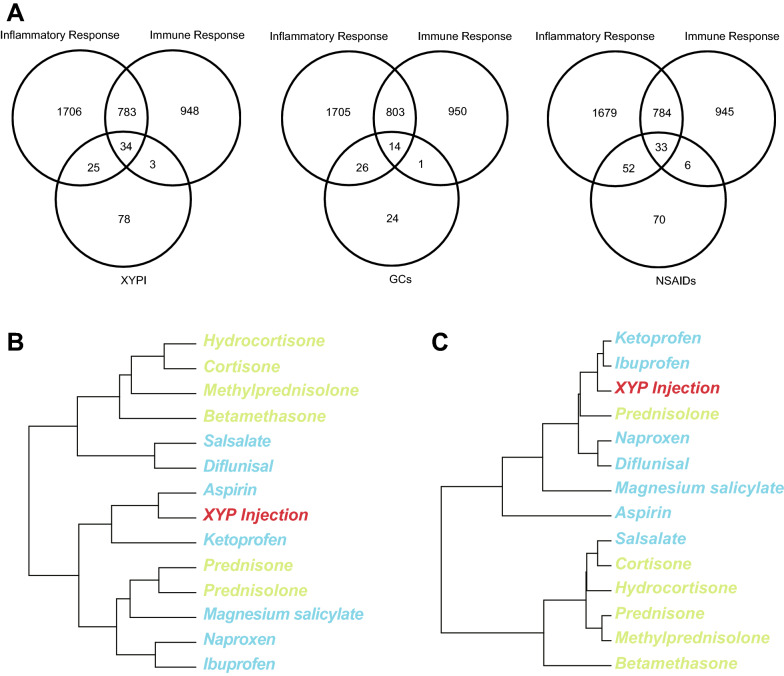
Comparison of targets and pathways of XYPI, GCs and NSAIDs. **A** Venn diagram of the drug targets of XYPI, GCs, and NSAIDs with genes in inflammatory and immune pathways. **B**, **C** Hierarchical clustering of XYPI (red), GCs (green) and NSAIDs (blue) based on pathway fingerprint similarity restricted to inflammatory (**B**) and immune (**C**) pathways

To explore the role of XYPI in immune and inflammatory responses, a Venn diagram was generated to compare the differences between XYPI drug targets and genes in the GO terms “immune response” and “inflammatory response”. The results showed that 59/140 and 37/140 targets of XYPI participate in immune and inflammatory responses, respectively. Similar results were obtained for GCs and NSAIDs (Fig. 2A). This result implies that most targets of XYPI, GCs and NSAIDs participate in immune and inflammatory pathways. Next, hierarchical clustering of XYPI, GCs and NSAIDs based on pathway fingerprints showed that XYPI was most similar to two NSAIDs (aspirin and ketoprofen), if the pathways were restricted to inflammation-related pathways (Fig. 2B). In addition, XYPI was most similar to ketoprofen and ibuprofen, when restricted to immune pathways (Fig. 2C). These results indicate that XYPI is more similar to NSAIDs in terms of immunity and inflammation. Hierarchical clustering based on pathway fingerprints shows that GCs and NSAIDs separate into different clusters, which indicates that GCs and NSAIDs have distinct target pathways in terms of inflammation and immunity.

### Predictions of drug-pathway associations of XYPI based on a “drug-target-pathway” heterogeneous network

**Fig. 3:**
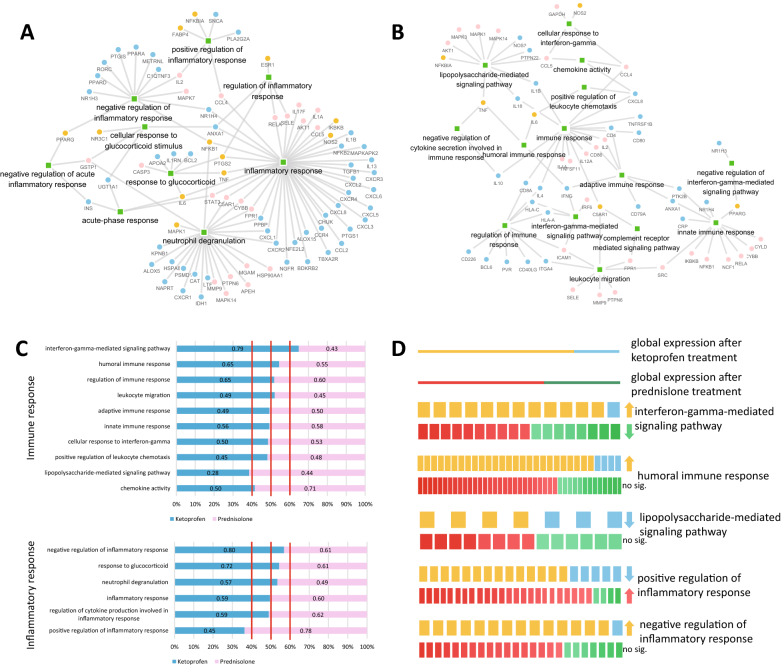
Comparison of XYPI and anti-inflammatory drugs in terms of inflammatory and immune pathways. **A** “drug-target-pathway” heterogeneous network of XYPI and NSAIDs in terms of inflammatory response. The green square was sharing pathway of XYPI and NSAIDs, and the circle was target of XYPI and NSAIDs. Blue circle was unique target of NSAIDs, pink circle for unique target of XYPI, and orange circle for common target of XYPI and NSAIDs. **B** “drug-target-pathway” heterogeneous network of XYPI and GCs in terms of immune response. The green square was sharing pathway of XYPI and GCs, and the circle was target of XYPI and GCs. Blue circle was unique target of GCs, pink circle for unique target of XYPI, and orange circle for common target of XYPI and GCs. **C** Expression of sharing pathway after Ketoprofen and Prednisolone treatment based on signature data from LINCS. The upregulated gene ratio was marked for each term. **D** Significantly differential expressing pathway after Ketoprofen and Prednisolone treatment. Upregulated genes or biological processes by Ketoprofen are marked with yellow, and downregulated ones are marked with blue. For prednisolone, upregulated genes or biological processes are marked with red, and downregulated ones are marked with green

To further illustrate the inflammatory and immune related characteristics of XYPI, we identified the shared pathways of XYPI and NSAIDs (ketoprofen) to predict the associated pathway of XYPI. In the inflammatory pathways, XYPI tends to affect molecules related to positive regulation of the inflammatory response and molecules involved in cytokine production, which include proinflammatory proteins such as NFKBIA, FABP4, IL2, and CCL4 (Fig. 3A). In the immune pathways, XYPI is more likely to be involved in leukocyte migration, the complement receptor signal transduction pathway, the LPS-mediated signaling pathway and the innate immune response. Among these factors, ICAM1, MMP9, PTPN6, and SRC are regulated by XYPI and participate in the process of leukocyte migration (Fig. 3B).

Transcriptome assays from the LINCS database also provide an opportunity to explore the expression of sharing pathway of reference drugs. Prednisolone and ketoprofen were selected as representative of GCs and NSAIDs, respectively, because of their high similarity with XYPI based on pathway fingerprints. In this study, differential expressing pathways after ketoprofen and prednisolone treatment were investigated based on LINCS data [22, 23] which can provide hint for XYPI regulation direction to pathway. We found that Ketoprofen and Prednisolone has distinct regulation for some pathways (Fig. 3C), including interferon gamma mediated signaling pathway, LPS mediated signaling pathway and regulation of inflammatory response (Fig. 3D). XYPI is more likely to be involved in Interferon γ-mediated signaling pathway similar to GCs, which was down regulated by prednisolone, but up regulated by ketoprofen based on the LINCS data (Fig. 3D). The sharing pathway between XYPI and ketoprofen, pro-inflammatory process (positive regulation of inflammatory response), was suppressed by ketoprofen, but anti-inflammatory process (negative regulation of inflammatory response) was activated by ketoprofen which imply that XYPI may have similar anti-inflammatory activity and mechanism with ketoprofen.

### Transcriptome of XYPI against LPS-activated murine macrophage model exhibiting its anti-inflammatory effect

**Fig. 4:**
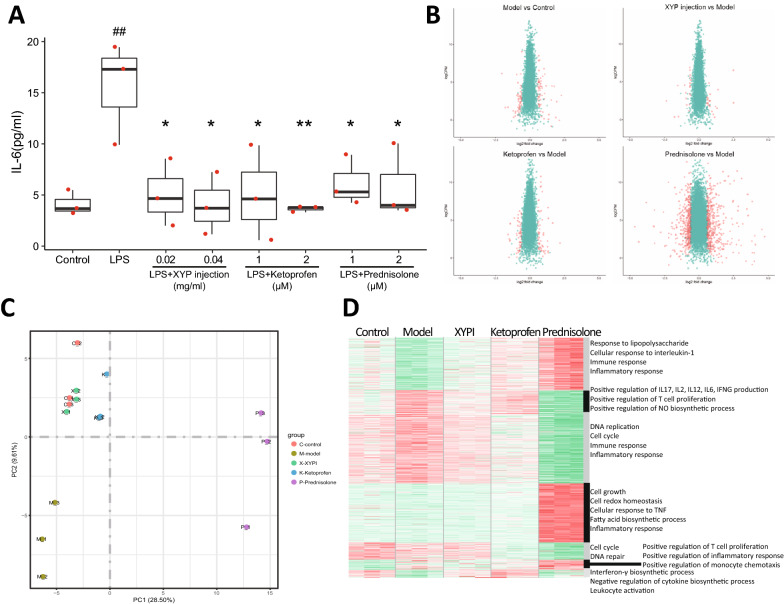
Transcriptome of XYPI pretreatment in an LPS-activated macrophage cell model. **A** Comparison of the anti-inflammatory effects of XYPI with those of ketoprofen and prednisolone. The effect of the three agents on expression of the inflammatory factor IL-6 induced by LPS in the supernatant of RAW264.7 cells after 24 h of pre-administration. * indicates the administration group vs the model group p <  = 0.05, ** p <  = 0.01. # indicates the model group vs the blank control group p <  = 0.05, ##p <  = 0.01. **B**–**D** Volcano plot (**B**), PCA (**C**) and hierarchical clustering (**D**) of the transcriptome of the control group, model group (LPS-induced macrophage model), and drug group (pretreatment with high-dose XYPI, high-dose ketoprofen and high-dose prednisolone)

To validate the prediction MoA of XYPI (similar to ketoprofen), and to compare the pharmacological effects of XYPI, an LPS-activated murine macrophage model was used. Prednisolone and ketoprofen were selected as representative GCs and NSAIDs, respectively, because of their high similarity with XYPI based on pathway fingerprints. In LPS-activated macrophages, transcription of the cytokine IL6 was increased after 48 h of stimulation (Fig. 4A). Pretreatment with low- and high-dose XYPI (low dose: 0.02 mg/ml, high dose: 0.04 mg/ml) strongly decreased cytokine expression in LPS-activated murine macrophages after 24 h of exposure. Pretreatment with ketoprofen and prednisolone (low dose: 1 µM, high dose: 2 µM) also reversed the upregulation of IL6. The results show that preadministration of XYPI has a significant anti-inflammatory effect by reducing IL-6 in activated macrophages, and this effect is similar to that of ketoprofen and prednisolone.

Transcriptome analysis based on RNA-seq was performed to detect the gene expression profile of LPS-activated macrophages after XYPI treatment, and ketoprofen and prednisolone were used as positive controls. A volcano plot of differentially expressed genes showed that the model group had a total of 117 differentially expressed genes compared with the control group, including 62 upregulated genes and 55 downregulated genes. Compared with the model group, the XYPI high-dose administration group had a total of 45 differentially expressed genes, including 23 upregulated genes and 22 downregulated genes. The ketoprofen high-dose group had 118 differentially expressed genes, including 55 upregulated genes and 73 downregulated genes. There were 883 differentially expressed genes in the prednisolone high-dose group, including 456 upregulated genes and 427 downregulated genes (Fig. 4B). The results of PCA in Fig. 4C show that the distance between the XYPI group and the control group was very small, indicating that after XYPI preadministration, the gene expression pattern of RAW263.4 cells was similar to that of the control group. As a positive control drug, ketoprofen had a gene expression pattern relatively similar to that of the control group. Although prednisolone has an anti-inflammatory effect, the gene expression pattern after treatment with prednisolone is very different from that of the control group and the group treated with XYPI and ketoprofen. The above results imply that XYPI may have a similar anti-inflammatory mechanism to ketoprofen, but it is quite different from the anti-inflammatory mechanism of prednisolone. A heatmap of hierarchical clustering was performed on the differentially expressed genes in each group, revealing a total of 7 distinct gene expression patterns, and functional enrichment analysis was performed for each group of genes (Fig. 4D), which showed that high-dose XYPI pretreatment reversed the downregulation of genes related to the response to LPS and the immune and inflammatory response and recovered the inflammatory response by suppressing the positive regulation of cytokine and NO biosynthesis processes and T cell proliferation (Fig. 4D). Compared with that of the model group, the response to LPS and IL1 was significantly upregulated after prednisolone treatment, and DNA replication, the cell cycle and immune and inflammatory responses were suppressed.

### Validation of drug-pathway associations of XYPI from transcriptome data

To predict the specific pathways regulated by XYPI, the shared metapath ‘drug-target-pathway-target-drug’ of the heterogeneous network was extracted for XYPI and NSAIDs. In Fig. 3A and B, possible drug-pathway association of XYPI from the “drug-target-pathway” heterogeneous network is shown in terms of the inflammatory response and immune response. The regulation direction to possible pathway of XYPI was validated based on transcriptome data for XYPI, ketoprofen and prednisolone.

**Fig. 5:**
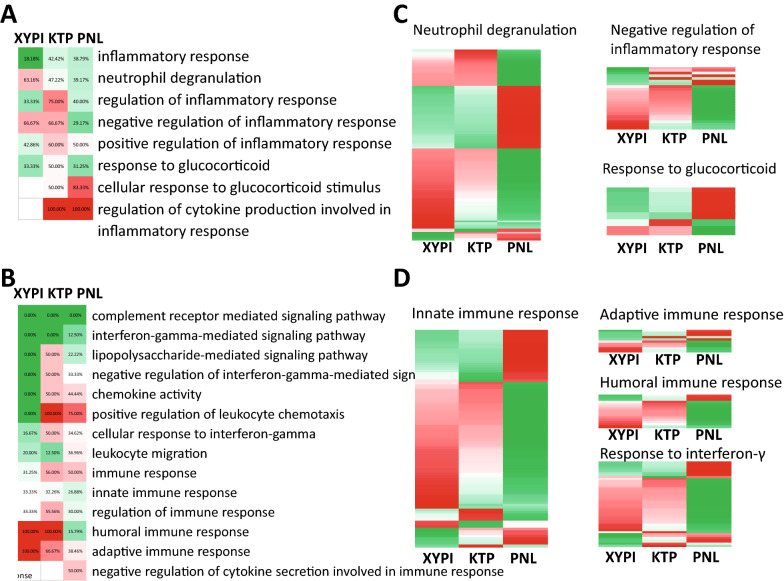
Expression of the shared pathways of XYPI, ketoprofen (KTP) and prednisolone (PNL). **A**, **B** Pathway activation and repression after drug treatment in terms of inflammatory (**A**) and immune pathways (**B**). **C**, **D** Heatmap of genes in the shared pathways after drug treatment in terms of the inflammatory (**C**) and immune pathways (**D**)

In terms of inflammatory response, Fig. 5A showed that neutrophil degranulation and negative regulation were upregulated by XYPI (63.1% of genes upregulated in the former and 66.7% in the latter), same as ketoprofen (47.2% of genes upregulated in the former and 66.7% in the latter) which is consistent with the prediction result “XYPI may have similar anti-inflammatory activity and mechanism with ketoprofen”. The heatmap of differential gene expression in the XYPI, ketoprofen and prednisolone pathways shows that most genes involved in neutrophil degranulation and negative regulation of the inflammatory response pathway were upregulated after XYPI and ketoprofen treatment but downregulated after prednisolone treatment (Fig. 5B). Genes related to ‘cellular response to glucocorticoid stimulus’ were obviously upregulated by prednisolone but downregulated by XYPI and ketoprofen (Fig. 5B). This implied that prednisolone had an expression pattern distinct from those of XYPI and ketoprofen in terms of the inflammatory pathway.

A comparison of the immune pathways for drug regulation (Fig. 5C) showed that the innate (61/91 genes upregulated), adaptive (5/10 genes upregulated) and humoral (12/15 genes upregulated) immune responses were activated by XYPI but repressed by prednisolone (32/91, 4/10, and 3/15 genes upregulated, respectively). In addition, “cellular response to IFN-γ” was upregulated by XYPI (29/45 genes upregulated). In previous prediction, XYPI is more likely to be involved in Interferon γ-mediated signaling pathway which was up regulated by ketoprofen. So XYPI and ketoprofen both activate the pathway related to IFN-γ, which was down regulated by prednisolone on the contrary. The heatmap of differential gene expression in the XYPI, ketoprofen and prednisolone pathways mentioned above shows that most genes involved in innate, adaptive and humoral immune responses and cellular responses to IFN-γ were upregulated after XYPI and ketoprofen treatment but downregulated after XYPI treatment (Fig. 5D). This implied that prednisolone repressed most aspects of the immune response.

These results imply that XYPI has similar regulation to immune and inflammatory pathway with ketoprofen, not prednisolone, which was predicted based on pathway fingerprints similarity evaluation. Second, drug-pathway associations of XYPI predicted by HIN were validated by the transcriptome data.

## Discussion

As commonly used anti-inflammatory drugs in the clinic, GCs and NSAIDs were selected as controls to study the anti-inflammatory and immune effects of XYPI. XYPI injection has similar targets as GCs because XYPI clustered with GCs in hierarchical clustering based on target similarity. However, XYPI has similar pathway fingerprints as NSAIDs. The transcriptome of the LPS-activated macrophage model after drug treatment shows that the gene expression pattern of macrophages after XYPI treatment is much more similar to that of ketoprofen, with no repression of the immune response, than to prednisolone. XYPI and ketoprofen both participate in the positive regulation of the inflammatory response and cytokine production. GCs are more likely to negatively regulate the signaling pathways mediated by interferon γ and are more involved in the function of acquired immunity. The expression pattern of these pathways after XYPI treatment is diametrically opposite that of GCs. We propose that XYPI may have a similar anti-inflammatory mechanism as NSAIDs.

These results indicate that pathway fingerprints provide a new approach for pathway based drug discovery. Even for a single drug compound, the mechanism should be explored from a multitarget perspective. A drug's multiple targets (direct and indirect) interact with each other to shut down a cellular pathway, which may be an unintended pathway, demonstrating the potential for polypharmacy to impact complex diseases. In addition to target profiles, pathway fingerprints have also been used in polypharmacological studies to describe the function of drug therapy [13, 15]. In this study, similarity analysis of pathway fingerprints and target profiles were both applied to investigate the MoA of natural products (XYPI). The experimental results confirmed that pathway fingerprints can be used to evaluate drug similarity and to predict MoA.

**Fig. 6:**
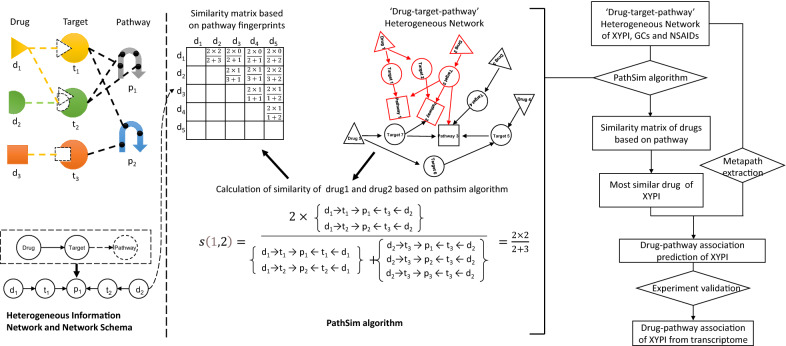
Workflow diagram of drug similarity evaluation based on pathway fingerprints. Left part shows network schema, meta-path, path instance of drug-target-pathway network. In the middle, the explanation of PathSim algorithm was shown using calculation of similarity of drug1 and drug2 as an example. First, based on the ‘drug-target-pathway’ Heterogenous network, metapath between drug1,2 and metapath for themselves were retrieved. Then the metapath is used for calculation of similarity between drug 1 and drug 2. Right part shows the workflow diagram of this study

Natural products from traditional medicine inherit bioactivity from their source herbs. However, the pharmacological mechanism by which they protect against disease is often unclear and studied insufficiently. The prediction of MoA based on structures and target profiles has been performed extensively in drug discovery. However, the “drug-target-pathway” heterogeneous network provides new insight into drug MoA. Pathway fingerprints extracted from a “drug-target-pathway” heterogeneous network can describe the pathways affected by drugs, which are meaningful combinations of direct and indirect targets of drugs. The similarity evaluation of pathway fingerprints based on recommendation systems used in social networks is a technological transformation of social network technology to pharmacological research. Compared to novel compounds, natural products have more easily predictable bioactivities based on traditional use. Pathway fingerprint similarity provides new insight into natural products compared with reference drugs, which are selected approved drugs with similar bioactivity. Natural products with similar pathway fingerprints may have similar MoA to approved drugs. In our study, pathway fingerprints of drugs can provide new ideas for drug similarity investigations. Similarity evaluation for heterogeneous networks based on recommendation systems for social networks provides good reference methodologies for pathway fingerprints (Fig. 6).

In summary, pathway fingerprints were applied to an XYPI MoA investigation. XYPI, GCs and NSAIDs have considerable anti-inflammatory effects. However, we found that XYPI has a similar target profile to GCs but similar pathway fingerprints to NSAIDs. Based on the experimental validation of the transcriptome, we found that the expression profile of ketoprofen is similar to that of XYPI, but prednisolone has a distinct profiling pattern, which indicates that the anti-inflammatory mechanism of XYPI may be different from that of GCs but similar to that of NSAIDs because XYPI does not have an immunosuppressive effect, unlike GCs. This experimental result is consistent with the computational prediction based on pathway fingerprints. This study used XYPI, an andrographolide derivative, as an example and proposed a new approach for investigating the pharmacological mechanism of natural products using pathway fingerprint similarity based on a “drug-target-pathway” heterogeneous network.

## Supplementary Information

**Additional file 1. **Hierarchical clustering of XYPI, positive and negative controls based on target similarity (A) and pathway fingerprint similarity (B).

**Additional file 2. **Hierarchical clustering of XYPI, GCs and NSAIDs based on pathway fingerprint similarity with different drug-target interaction cutoff (0.3-0.5) using three types of pathway datasets(GO, Reactome, WikiPathways).

## Data Availability

The code is available at GitHub (https://github.com/huihui1126/drugSim-pathway). The transcriptome data are available at SRA BioProject database (https://www.ncbi.nlm.nih.gov/bioproject/714798).
