# Supplementary material for: Investigation of pharmacological mechanism of natural product using pathway fingerprints similarity based on “drug-target-pathway” heterogenous network
Source: J Cheminform. 2021 Sep 20;13:68. doi: 10.1186/s13321-021-00549-5 (PMC8454151; doi:10.1186/s13321-021-00549-5)
Supplement: Supplementary file 1 — Additional file 1. Hierarchical clustering of XYPI, positive and negative controls based on target similarity (A) and pathway fingerprint similarity (B). [file 13321_2021_549_MOESM1_ESM.pdf]

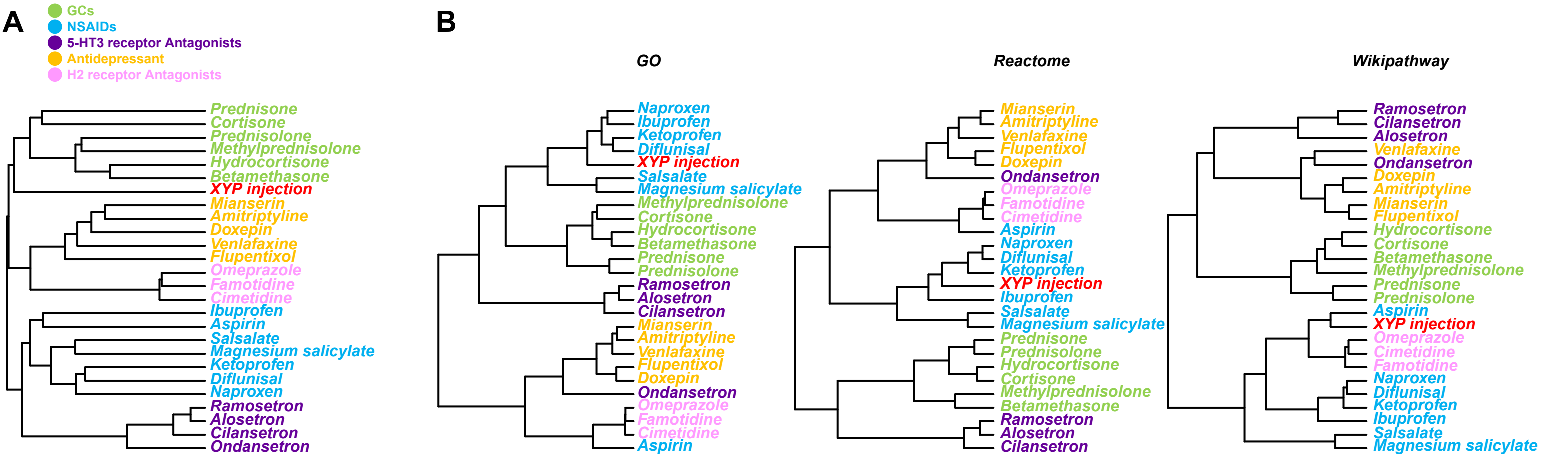
A
GCs
NSAIDs
5-HT3 receptor Antagonists
Antidepressant
H2 receptor Antagonists
Prednisone
Cortisone
Prednisolone
Methylprednisolone
Hydrocortisone
Betamethasone
XYP injection
Mianserin
Amitriptyline
Doxepin
Venlafaxine
Flupentixol
Omeprazole
Famotidine
Cimetidine
Ibuprofen
Aspirin
Salsalate
Magnesium salicylate
Ketoprofen
Diflunisal
Naproxen
Ramosetron
Alosetron
Cilansetron
Ondansetron
B
GO
Reactome
Wikipathway
