# Supplementary figures and images for: Investigation of pharmacological mechanism of natural product using pathway fingerprints similarity based on “drug-target-pathway” heterogenous network
Source: J Cheminform. 2021 Sep 20;13:68. doi: 10.1186/s13321-021-00549-5 (PMC8454151; doi:10.1186/s13321-021-00549-5)

0.3

0.4

0.5

GO

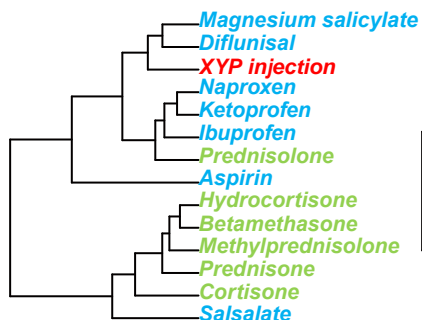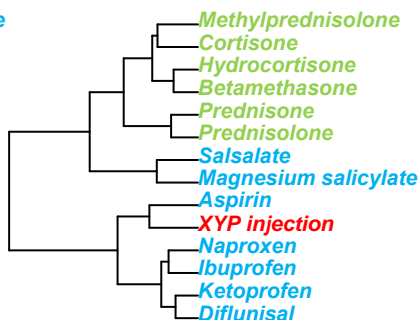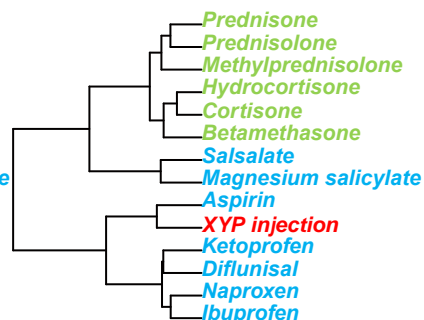

Reactome

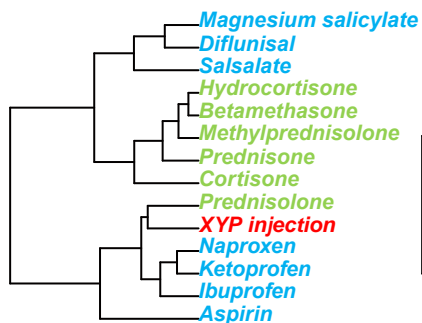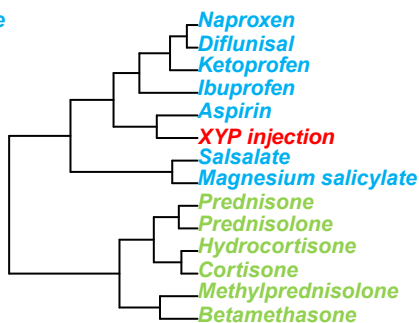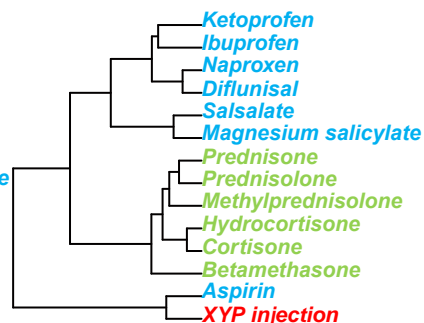

Wikipathway

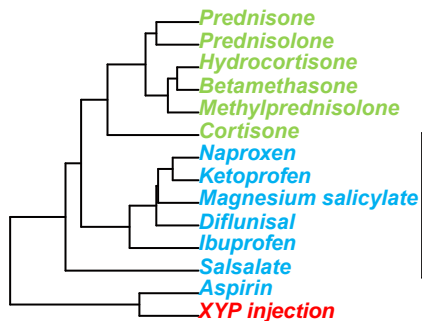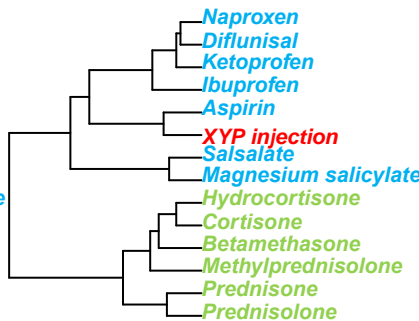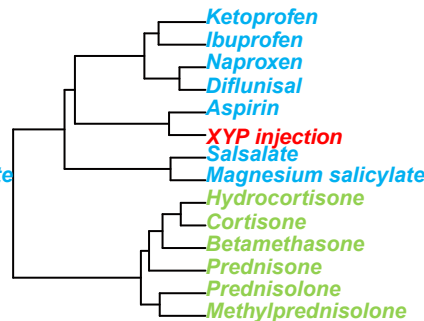

Supplement: Supplementary file 2 — Additional file 2. Hierarchical clustering of XYPI, GCs and NSAIDs based on pathway fingerprint similarity with different drug-target interaction cutoff (0.3-0.5) using three types of pathway datasets(GO, Reactome, WikiPathways). [file 13321_2021_549_MOESM2_ESM.pdf]
